# Peer Review of “Development and Content Validity of the Handwashing Index (Preprint)”

**DOI:** 10.2196/67587

**Published:** 2024-11-06

**Authors:** Vanessa Fairhurst, Monira Sarmin, Sayan Mitra, Olajumoke Ope Oladoyin, Paul Hassan Ilegbusi, Toba Olatoye, Katherine McNeill

**Affiliations:** 1 PREreview, Portland, OR, United States; 2 International Centre for Diarrhoeal Disease Research, Dhaka, Bangladesh; 3 University of Sydney, Sydney, Australia; 4 University of Texas Health Science Center at Houston, Houston, TX, United States; 5 Ondo State College of Health Technology, Akure, Nigeria; 6 University of Ilorin, Ilorin, Nigeria

**Keywords:** handwashing, index, hygiene, infection control, scale

*This is the peer-review report submitted for the preprint “Development and Content Validity of the Handwashing Index.”*

This review is the result of a virtual collaborative live review discussion organized and hosted by PREreview and JMIR Publications on September 27, 2024. The discussion was joined by 20 people: 2 facilitators, 2 members of the JMIR Publications team, 2 authors, and 14 live review participants, including 2 who agreed to be named (but did not help compile this final review): Mitchell Collier and Goktug Bender. The authors of this review have dedicated additional asynchronous time over the course of 2 weeks to help compose this final report using the notes from the live review. We thank all participants who contributed to the discussion and made it possible for us to provide feedback on this preprint.

## Summary

The study [1] aims to develop and validate a tool for measuring the frequency of handwashing behavior to improve hygiene practices. Such practices are critical for preventing diseases (especially those transmitted via fecal-oral or nasal routes), and as seen during the COVID-19 pandemic, handwashing plays a crucial role in the prevention of infection. The primary goal of the study was to create a handwashing index (HWI) to effectively track handwashing frequency habits. The researchers adapted the Physical Activity Questionnaire for Adolescents to develop the Handwashing Questionnaire (HWQ) and used a structured 4-stage process, following COSMIN (Consensus-Based Standards for the Selection of Health Measurement Instruments) guidelines, for its development and validation. The authors successfully developed and validated this HWI, employing a well-grounded study plan and methodology. The 4-stage process was clearly described, providing a solid foundation for their research.

The questionnaire initially contained 6 questions, which were refined down to 4 that effectively assessed handwashing practices. These questions were validated using expert input, yielding a strong content validity index (CVI) of 0.9. The validated HWI can categorize individuals into groups like regular and nonregular hand washers, as well as decliners, and offers a simple, practical, and effective tool for tracking handwashing behavior. The conclusions are generally supported by the data, as the content validity results align with the feedback from expert judges. In addition to the validation process, the research also yielded some findings of its own, such as the fact that compliance with regular handwashing was not as high as expected post pandemic, based on participant self-reporting.

The HWI can be applied across different settings, including health care, public health campaigns, and even individual self-monitoring. Its simplicity makes it suitable for both resource-rich and resource-limited environments. Actual items of the HWI are presented clearly, along with instructions for scoring, making it straightforward for others to reproduce the questionnaire. Reviewers found the novel approach to assessing handwashing practices particularly interesting. They noted that this methodology could be adapted to other behavior-monitoring surveys that may require contextual adjustments. Although the HWI does not assess the method of handwashing, it effectively categorizes compliance with general frequency recommendations. This index could serve as a valuable tool for health professionals and public health officials to track and promote better hand hygiene, especially in low-resource settings.

## Major Concerns and Feedback

Alongside its strengths, there exist several ways the study and its reporting could be improved, in areas such as scope and analysis, the survey tool’s reproducibility in diverse settings, and clarity in definitions and other aspects. Such improvements would greatly benefit the global community.

### Concerns With Techniques/Analyses

The Methods section would benefit from more elaboration of the procedure, which will help others reproduce the same research.

#### Sampling

- The validation process was conducted with only a small sample size of 57 health professionals from Ghana, which could introduce selection bias and limit the generalizability of the findings to the general global population. It is uncertain whether a survey instrument validated for 1 research purpose can be modified for an entirely different purpose.
- The convenience sampling approach raises concerns about selection bias, although the authors acknowledge this. Some data indicate that the sample may not be representative (eg, trends in age by gender—is the general age of male health care workers half that of females? Or did the study fail to recruit more experienced men?).
- It would be helpful to specify how many participants were invited versus how many completed the study to provide context on participation rates.
- The inclusion of only health professionals limits the applicability of the tool to a general population (and contradicts the stated aim to be used in this broad manner).

#### Scope

- The only aspect of handwashing measured by the questionnaire is frequency. It does not study handwashing methods (eg, duration, movements, soap used, drying equipment); yet the method by which handwashing is performed can dramatically impact its effectiveness. This limits how well this index can measure compliance with recommended practice, assess hygiene performance, and be an improvement upon existing guidelines (eg, World Health Organization).
- Given this limit in scope, the authors should make this focus on frequency much more prominent throughout the article (the word “frequency” appears only 3 times). This important qualifier could be done in places such as the name of the index itself (eg, handwashing [frequency] index), the abstract (eg, “There is no validated scale to measure [the frequency of] this habit”), and the conclusion (eg, “Interventions aimed at improving [the frequency of] handwashing in the global community”).

#### Measurement

- The cutoff point for item scoring appears too low, as a score of 4 is categorized as regular practice, despite the reported highest score being 20. Furthermore, the upper limit frequency is relatively low, limiting generalizability. This should be reconsidered, and the response options recategorized to enhance clarity.
- The use of standard scales (eg, Likert scale) would be an improvement.
- The authors should confirm if “average” is the appropriate descriptive statistical measure, or if “median” would be more suitable for the responses.
- The authors provide categories for handwashing behaviors, such as “regular,” “non-regular,” and “decliners,” but they do not explain whether the thresholds for these categories are empirically derived or based on expert consensus. Furthermore, the term “decliners” might carry a negative connotation (and thus be underreported) and could be replaced with a more neutral descriptor, such as “low-frequency hand washers.”
- There is no clear indication that the psychometric properties of the survey instrument were tested, despite mentioning a validation process involving 10 expert judges. This limitation could affect the robustness of the findings.
- The study is based on self-reported data, which may introduce response bias. The participants might overestimate or underestimate their handwashing behavior. For example, participants may report more frequent handwashing than they actually practice, especially because hand hygiene is a socially desirable behavior. No specific controls are in place to mitigate this bias.

#### Clarity of Instrument

The following concerns should be considered regarding the clarity of the instrument, as responses may vary from what is intended if aspects are not clear to respondents:

- The questionnaire contained not only a single-choice selection but also a rating scale used alongside this. Reviewers were unsure of the reasoning of this or how it would be answered accurately (since it could be unclear to participants).
- There is no definition of handwashing (eg, how to do it and how long to do it for). All participants may not have had the same definition of washing their hands in mind when responding.
- It is unclear what the difference is between “active handwashing” and “regular handwashing.” Moreover, even if the authors have a meaningful difference in mind, it is unlikely to be clearly understood by respondents, and the subjective interpretation of “active” was up to the respondents. Thus, the author statement that the index measures “how active individuals practice” handwashing is insufficiently substantiated.
- The authors should define for respondents “hardly ever,” “sometimes,” and “quite often,” to avoid ambiguity. Even if definitions were provided, it is not clear why there is a hybrid between the other quantitative measures and this qualitative one (and the mixture/nonparallel format could be confusing to respondents). The authors might consider changing these response options to quantitative measures, in parallel with the other questions.

### Details for Reproducibility of the Study

- The tool’s reproducibility could be improved by including more detailed participant selection criteria and clearer descriptions of the handwashing behavior scoring methodology.
- The authors do not discuss how the thresholds for defining handwashing categories were derived, which introduces subjectivity and may limit the tool’s reliability.
- The potential for cultural differences in handwashing behaviors impacting the tool’s applicability across populations is not discussed, which could be another limitation for generalizability.
- Greater clarity on how to answer the questionnaire would improve reproducibility, including a definition on what exactly counts as handwashing, and the difference between active and regular handwashing.
- Data are not openly available. While the article indicates that data are “available upon reasonable request,” this has been proven to be a highly unreliable method of access (particularly with the qualifier of “reasonable”). Furthermore, data that are not professionally curated are less likely to be usable long-term).
- The tool could require some improvement in terms of making it reproducible in other settings (eg, clarity in definitions) in order to be more useful to a global community.

### Figures, Tables, and Results

Recommendations for improvement: To enhance clarity and readability, the following adjustments should be considered:

- The tables in the study are generally clear and well-labeled, displaying key data, such as participant demographics, scoring categories, and CVI calculations, in an easy-to-understand format. However, including more detailed legends or footnotes for the CVI calculations, participant scoring, and definitions could improve clarity, especially for readers unfamiliar with the validation process.
- The authors should ensure consistent formatting throughout tables and figures, including the ability to view table numbers and titles when in the detail view and the proximity of the tables to the descriptions. Such changes could better enable readers to follow the tables along with the text.
- The addition of graphs could provide a good visual comparison of the scores.
- Table 1 (sociodemographic key): While generally clear, this table should include a key or legend explaining the differences between a certificate, diploma, and degree.
- Table 2: (clarify “Active” handwashing): As mentioned above, the manuscript should specify what “active” refers to (vigor of handwashing, respondent’s daily activity level, or something else).
- Table 3: The authors should add a footnote describing the calculation of “I-CVI” and “S-CVI.”
- Tables 3 and 4 (editing and abbreviation clarification): The manuscript should provide more detailed footnotes explaining the abbreviations used.
- Table 4: While generally concise and straightforward, it could benefit from highlighting the final accepted items, especially for readers who want to focus on the validated items at a glance. A brief legend explaining why “item 3” was removed from earlier versions would be helpful.
- Include a Results section: The study lacks a dedicated Results section, which is essential for presenting findings.

### Limitations Discussed

The conclusions are largely supported by the data, as the content validity results and expert feedback align with the development and validation of the HWI. The authors appropriately acknowledge many limitations of the study, including the small sample size, the use of self-reported data, and the need for further validation. Yet the conclusion could discuss these limitations in a more nuanced manner and more explicitly acknowledge how they may affect the generalizability and robustness of the findings. Incorporating these caveats would make the conclusions more balanced. Many important limitations go unmentioned, including:

- At this stage, the HWI is only validated for content, and its effectiveness as a general tool has not yet been fully established.
- The potential impact of cultural differences on handwashing behaviors, which may affect the reproducibility and applicability of the index in different populations.
- The reasons behind the selection strategy, and biases that might arise from it, should be acknowledged more clearly.
- The important limitation of measuring only frequency, and not different behaviors when washing hands (eg, length of time, soap, what was used to dry hands). Such statements are particularly important given the claim that this index can improve public health (whereas much of the literature cited in the Introduction is based on measures of quality beyond frequency).
- Authors could detail their further recommendations regarding future validation studies.

### Ethics

- Ethical implications arise from the study, particularly regarding poststudy education on handwashing for participants identified as “decliners” or “non-regulars,” especially given their role in health care settings.
- The authors should clarify how they communicated with participants. This includes ensuring that participant involvement was not framed in a way that could be perceived as judgmental or evaluative of their professional practice, and that their participation (or not) in this study would not at all impact their status at their job. This would ensure ethical integrity and transparency in the research process.
- Although the study mentions following the protocols of the Ghana Health Service Review Committee, more details about the ethical approval process would strengthen transparency.

## Minor Concerns and Feedback

### Clarification of Terminology, Acronyms, and Descriptions

- The authors use the key terms “HWQ,” “HWQ-I,” and “HWI” throughout the manuscript. If each of these terms is indeed important and distinct, the authors should at the outset clearly define each of these terms and explain the distinction, given their similarity. Furthermore, they should introduce the formal name of the proposed standard index more clearly and explicitly. Thereafter, each term should be used consistently throughout the manuscript to prevent confusion.
- The manuscript requires clearer definitions of key acronyms at first use; for example, HWQ is used in the first paragraph, but the acronym is not defined until the Methods section.
- The authors should provide a definition of handwashing in general. If this ambiguity (including toward respondents) was deliberate, this should be explicitly stated.
- The meaning and differences of terms such as “S-CVI” and “UA” should be explained more thoroughly. Similarly, CVI calculations and participant scoring need clearer descriptions, especially in Table 3.
- The authors’ use of the term “monitor” (eg, “HWI demonstrated excellent content validity, showing its relevance for monitoring handwashing”) is not appropriate in this setting, as monitoring indicates a third party overseeing/verifying behavior.

### Other

- The authors should organize the Introduction to provide context, clearly state the research questions, and outline the study’s objectives.
- The main title and short title are mismatched (“HWI” vs “scale”).
- Further clarification is required on scoring methodologies.
- The method of survey administration should be stated in the manuscript (eg, paper, website).

By addressing these concerns, the manuscript will become more readable, comprehensive, and effective in conveying the study’s findings and objectives.

### Suggestions for Future Work

- A follow-up study could delve deeper into handwashing practices by assessing respondents’ perceived adherence to handwashing protocols (ie, the method—and not just frequency—of handwashing). This could measure aspects such as duration, availability of soap/water to the respondents, etc.
- Conducting observational research or surveys to verify the respondents’ actual handwashing techniques would help to avoid self-reporting bias.
- While the conclusion reflects the study as designed, before approaching the global community, it needs local validation, a greater sample size, and diverse participants across the general population (beyond health care professionals, who we assume practice handwashing better than the general population).
- Given the sample population of health care workers, future work could include modifying the questionnaire to focus specifically on that population. The instrument could add questions specific to a health care environment, including the environment itself and the characteristics of the respondent (eg, their role in health care). Such changes could make it even more applicable to health care workers.

## Abbreviations

COSMIN: Consensus-Based Standards for the Selection of Health Measurement Instruments
CVI: content validity index
HWI: handwashing index
HWQ: Handwashing Questionnaire
